# Correction to “Glycoengineered extracellular vesicles released from antibacterial hydrogel facilitate diabetic wound healing by promoting angiogenesis”

**DOI:** 10.1002/jev2.70314

**Published:** 2026-05-28

Wang, K., He, Q., Yang, M., Qiao, Q., Chen, J., Song, J., Zang, Na., Hu, H., Xia, L., Xiang, Y., Yan, F., Hou, X., & Chen, L. (2024). Glycoengineered extracellular vesicles released from antibacterial hydrogel facilitate diabetic wound healing by promoting angiogenesis. *Journal of Extracellular Vesicles* 13: e70013. https://doi.org/10.1002/jev2.70013

In the s‐MSC panel of Figure 2e, the image for CD73 was inadvertently duplicated from the CD105 panel during figure assembly. The corrected Figure 2 is shown below. The online version of the article has been corrected.

**FIGURE 2:**
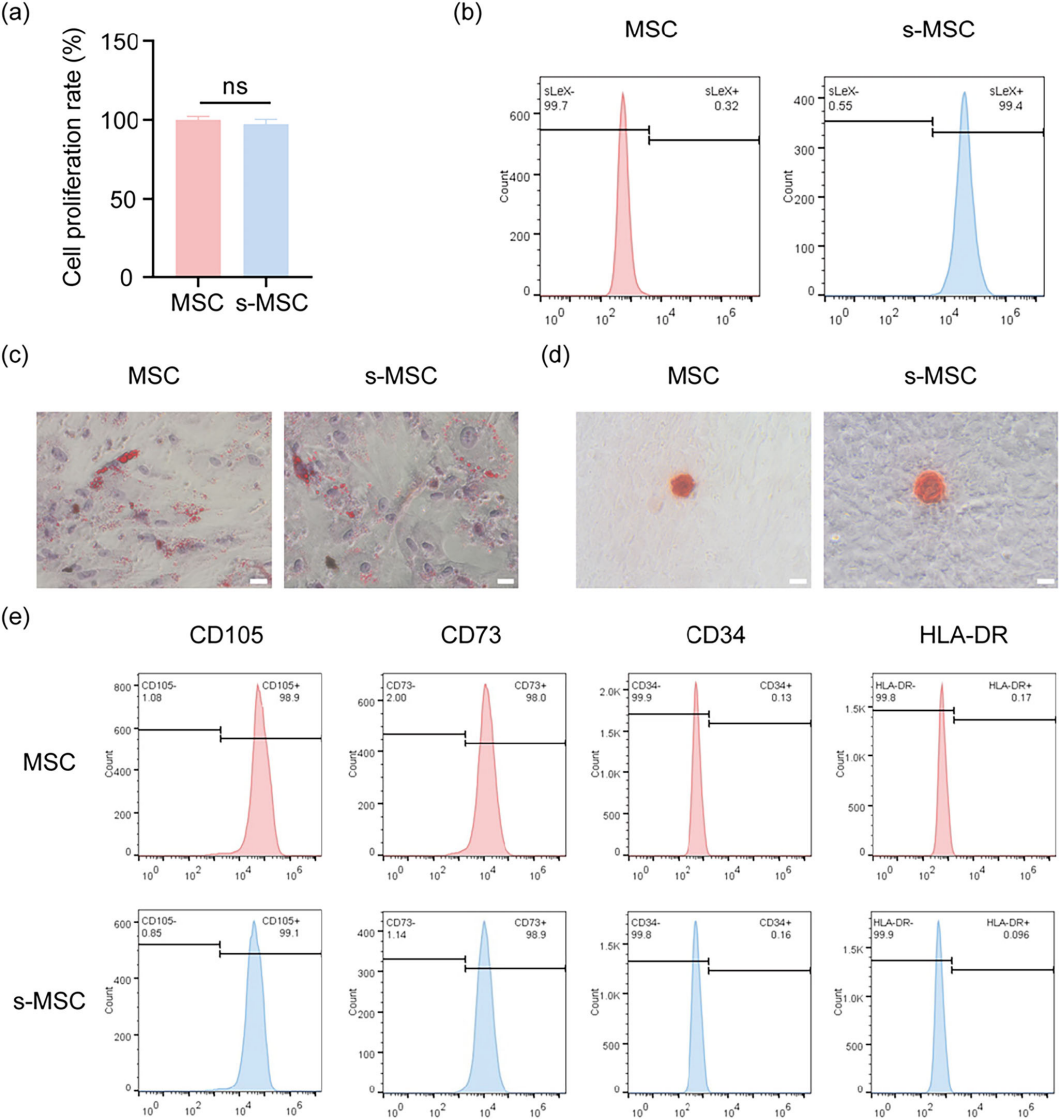
Identification of MSCs and s‐MSCs. (a) Cell viability assay of MSCs and s‐MSCs. (b) Detection of sLeX expression on s‐MSC surface by flow cytometry. (c)–(d) Oil Red O and Alizarin Red staining of MSCs and s‐MSCs following adipogenic and osteogenic induction. Scale bar: 20 µm. (e) Identification of MSCs and s‐MSCs by flow cytometry. Data are shown as the mean ± SEM. ns, no significant difference; s‐MSCs, MSCs overexpressing sLeX.

We apologize for this error.

